# Eco-Friendly Feed Formulation and On-Farm Feed Production as Ways to Reduce the Environmental Impacts of Pig Production Without Consequences on Animal Performance

**DOI:** 10.3389/fvets.2021.689012

**Published:** 2021-07-06

**Authors:** Francine de Quelen, Ludovic Brossard, Aurélie Wilfart, Jean-Yves Dourmad, Florence Garcia-Launay

**Affiliations:** ^1^PEGASE, INRAE, Institut Agro, Saint-Gilles, France; ^2^SAS, INRAE, Institut Agro, Rennes, France

**Keywords:** life cycle assesment, multiobjective formulation, local feed ingredients, low-impact feed, pig fattening

## Abstract

Animal feeding has a major contribution to the environmental impacts of pig production. One potential way to mitigate such effects is to incorporate an assessment of these impacts in the feed formulation process. The objective of this study was to test the ability of innovative formulation methodologies to reduce the impacts of pig production while also taking into account possible effects on growth performance. We compared three different formulation methodologies: least-cost formulation, in accordance with standard practices on commercial farms; multiobjective (MO) formulation, which considered feed cost and environmental impacts as calculated by life cycle assessment (LCA); and MO formulation, which prioritized locally produced feed ingredients to reduce the impact of transport. Ninety-six pigs were distributed between three experimental groups, with pigs individually weighted and fed using an automatic feeding system from 40 to 115 kg body weight. Based on the experimental results, six categories of impacts were evaluated: climate change (CC), demand in non-renewable energy (NRE), acidification (AC), eutrophication (EU), land occupation (LO), and phosphorus demand (PD), at both feed plant gate and farm gate, with 1 kg of feed and 1 kg of live pig as functional units, respectively. At feed level, MO formulations reduced CC, NRE, AC, and PD impacts but sometimes increased LO and EU impacts. These formulations reduced the proportion of cereals and oil meals into feeds (feed ingredients with high impacts), while the proportion of alternative protein sources, like peas, faba beans, or high-protein agricultural coproducts increased (feed ingredients with low impacts). Overall, animal performance was not affected by the dietary treatment; because of this, the general pattern of results obtained with either MO formulation at farm gate was similar to that obtained at feed level. Thus, MO diet formulation represents an efficient way to reduce the environmental impacts of pig production without compromising animal performance.

## Introduction

Livestock production is a significant contributor to global environmental change. The associated greenhouse gas emissions, water pollution, acidification (AC), and primary energy consumption can have serious environmental impacts, in particular in territories with high concentrations of livestock (1, 2). For pig farming, such impacts are the consequences of feed production, direct farm energy use (electricity, gas, and oil consumption), and emissions from housing and manure management systems (3, 4). In particular, depending on the production system in question, animal feed accounts for 55–75% of the effects of climate change (CC), 70–90% of energy use, and 85–100% of land occupation (LO) associated with production (5). This is due, in part, to crop production processes for feed ingredients that are reliant on mineral fertilizers and pesticides, contribute to LO and transformation, consume significant amounts of energy, and use large-scale transportation networks (6). The challenge, then, is to reduce emissions and increase the efficient use of resources. Feed ingredients can vary dramatically in their environmental impacts; certain ingredients, like imported soybean meal, are resource intensive compared with alternative protein sources that can be locally produced (peas, faba beans, or high-protein agricultural coproducts) (6). Several studies have investigated the possibility of reducing the environmental impacts of pig production by modifying the composition of the diet. For example, Eriksson et al. (7) substituted soybean meal with peas and rapeseed meal in growing–finishing pig diets and observed reductions of 10% in energy use, 7% in global warming potential (GWP), and 17% in eutrophication (EU). Similarly, van Zanten et al. (8) showed that replacing soybean meal with rapeseed meal in the diets of finishing pigs reduced GWP as well as LO and energy use. A study of pig diets that substituted coproducts of wheat for corn and soybean meal also reported decreases in the potential for AC and EU, non-renewable energy (NRE) use, and GWP (9). Therefore, there is a possibility to reduce environmental impacts by selecting feed ingredients with relatively low impacts like alternative protein sources (peas, faba beans, or high-protein agricultural coproducts) or by using ingredients locally produced in order to reduce the impact of transport (6).

The traditional approach to feed formulation is based only on cost and makes no consideration of environmental factors. To reduce the overall impacts of pig production, new methods have been proposed that incorporate the environmental impacts of feed ingredients in the feed formulation process. For example, Garcia-Launay et al. (10) developed a multiobjective (MO) formulation method based on the environmental impacts of feed ingredients as calculated by life cycle assessment (LCA). Previous studies that have included environmental objectives in the calculation of feed formulations (9–11) have generated diets with lower proportions of cereals and oil meals and higher proportions of alternative protein sources (peas, faba beans, or high-protein agricultural coproducts). However, these studies were all based on models that assumed animal performance would be unaffected by these dietary changes. In general, feed formulations that are designed to minimize environmental impacts contain a higher proportion of protein-rich crops and coproducts, which may have potentially undesirable consequences with respect to the nutritional composition of feed and/or variability in energy, fiber, or protein content (12, 13). Indeed, using an experimental approach, Shaw et al. (14) reported a negative effect on pig growth of the incorporation of wheat middlings in the diet. Similarly, the replacement of soybean meal with rapeseed meal in the diet may also decrease pig performance (15). An increased incorporation of coproducts associated with the MO formulation could therefore adversely affect the pig performance and, consequently, reduce the improvement obtained at feed level. The objective of this study was then to test the effectiveness of innovative formulation methodologies in fattening pigs to reduce the environmental impacts of pig production, while taking into account their possible effects on animal performance. The global approach adopted was (i) to formulate diets based on these innovative feed formulation methodologies combining economy and environment, (ii) to test these diets experimentally on growing–finishing pigs, and (iii) to use the results of the experiment to assess the associated environmental impacts using LCA.

## Materials and Methods

Three different formulation methodologies were compared:

- least-cost formulation (Control-diet), in accordance with standard practices on commercial farms;
- MO formulation (Eco-diet) that simultaneously optimized feed cost and environmental impacts as calculated by LCA; and
- MO formulation using locally produced feed ingredients (Local-diet) to reduce the impact of feed transport.

### Feed Formulation

Information on the nutritional composition of feed ingredients was obtained from the French nutritional table INRA-CIRAD-AFZ (16). Diets were formulated to meet the nutritional requirements of an average growing [40–65 kg body weight (BW)] or finishing (65–115 kg BW) pig. Minimum contents for standardized ileal digestible amino acids were set according to expected performance using the InraPorc® model (17) and also took into consideration French regulatory guidelines on maximum feed protein content (18). Minimum and maximum values of net energy content were defined in accordance with NRC 2012. The ingredients used in this study were analyzed before the diets were created in order to adjust diet composition according to the real nutritional values of ingredients (dry matter, organic matter, crude protein, and gross energy). Consequently, the incorporation rates of wheat, corn, soybean meal, and sugar beet pulp were slightly modified because their energy and protein contents were slightly different from the ones in the tables. Ingredient prices and availability were provided by IFIP (Didier Gaudré, personal communication). Ingredient prices of October 2018 (price of wheat: 203€/t; price of soybean meal: 351€/t) were used.

The environmental impacts of ingredients were taken from the ECOALIM dataset (version 7, October 1, 2019, https://www6.inrae.fr/ecoalim/) of the AGRIBALYSE database (6). They included International Reference Life Cycle Data System (ILCD) metrics of AC potential (expressed in molc H^+^-eq/kg) and CC, which included land use change (CC, expressed in kg CO_2_-eq/kg). They also included Center for Environmental Studies (CML) EU potential (expressed in kg ${\text{PO}}_{4}^{3-}$-eq/kg from the Center for Environmental Studies); cumulative energy demand 1.8 (CED v1.8) as NRE demand (expressed in MJ/kg); and CML LO (expressed in m^2^year/kg) and phosphorus (P) demand (6) (PD; expressed in kg P/kg). For crops, data used for the life cycle inventories (crop management practices; yields; and amounts of fertilizers, pesticides, and seeds) were obtained from French agricultural data and represented the national averages. All impacts from the ECOALIM dataset were considered to be those at the storage agency gate for the least-cost formulation (Control-diet) and the Eco-diet formulation, and to be those at the farm gate for the Local-diet formulation (except for rapeseed meal and for amino acids, premix, and phytase, which are not assumed to be produced on farm). An economic allocation approach was used to partition environmental impacts between a product and its c-product (Supplementary Table 1) as described in Wilfart et al. (6, 19) and advised by the Food and Agriculture Organization (20) and the French guideline to perform environmental assessment of agricultural product (21). Furthermore, Ardente and Cellular (22) recommended the use of the economic allocation concerning production of a main product with high economic value where coproducts are only a side effect of production.

The MO method developed by Garcia-Launay et al. (10) was used to formulate the experimental diets. This method considers animals' nutritional requirements, the cost of feed, and various environmental impacts. A detailed description of the method can be found in the original publication (10). As developed, the MO formulation method uses linear programming (Simplex algorithm) in the Python programming language (http://www.python.org). For the least-cost formulation, only feed cost was minimized. For the MO formulation, the objective function (Equation 1) included global environmental impacts calculated through the LCA, i.e., CC, NRE, LO, and PD, under a varying constraint ϵ of maximum feed cost (Equation 2) (10). Constraints were added on the environmental impacts of the formulated feed to ensure that the MO formulation did not increase any impact by more than 5% relative to the environmental impacts of the reference least-cost feed (Equation 3). Constraints were also applied to nutritional composition (Supplementary Table 2) and the incorporation rates of feed ingredients (Equation 4) (Supplementary Tables 3–5).

(1) $$\begin{array}{l} f\left(x\right)=\underset{i\in I}{\sum }coef_{i}\frac{{Impact_{i}}^{t}x-Min_{i}}{Ref_{impact_{i}}-Min_{i}} \end{array}$$

(2) $$\begin{array}{l} c^{t}x\leq \epsilon \;\epsilon =\left\{Ref_{price},\ldots ,Max_{price}\right\} \end{array}$$

(3) $$\begin{array}{l} {Impact_{i}}^{t}x\leq 1.05\times Ref_{impact_{i}} \end{array}$$

(4) $$\begin{array}{l} \left(\begin{array}{c} q_{\min}\\ n_{\min}\\ 1 \end{array}\right)\leq \left(\begin{array}{c} Q\\ N\\ 1^{t} \end{array}\right)x\leq \left(\begin{array}{c} q_{\max}\\ n_{\max}\\ 1 \end{array}\right) \end{array}$$

$$\begin{array}{l} i=\left\{CC,\;NRE,\;LO,\;AC,\;EU\right\} \end{array}$$

${\text{Impac}{\text{t}}_{\text{i}}}^{t}$: vector of impact i of feed ingredients; c: matrix of feed ingredient prices; Max_price_: price of feed when formulating without constraint ϵ; Min_i_: level of impact i when formulated at lowest impact i; x: matrix of incorporation rates of feed ingredients (decision variables); *Ref*_*impac*_*t*__*i*__ and Ref_price_: impact i and price of least-cost feed formulation; coef_i_: weighting factor of impact I, with coefCC being double that of the other impacts. q_min_ and q_max_ are the minimum and maximum incorporation constraints on feed ingredients, respectively. n_min_ and n_max_ are the lower and upper bounds, respectively, for the nutritional constraints applied to the feed. The objective function weighted the environmental impacts of CC by 2, and those of NRE, LO and PD by 1.

The best feed formula is that for which the marginal decrease in the environmental index $(\frac{Impact_{i}^{t_{x}}}{Ref_{impact_{i}})}$ is less than the marginal increase in the cost index $\frac{c^{t}x}{Ref_{price}.}$

The Local-diet was also formulated with the MO approach but with locally produced ingredients (cereals and protein-rich crops like peas and faba beans) as well as rapeseed meal.

The composition and the environmental impacts of the three growing diets and the three finishing diets are given in Tables 1, 2, respectively.

**Table 1:** Composition of experimental growing diets^*a*^.

**Diets**	**Control-diet**	**Eco-diet**	**Local-diet**
**Ingredients, %**			
Corn	19.20	31.00	10.70
Wheat	36.00	15.22	29.50
Triticale	10.00		10.00
Barley	5.50		12.25
Wheat middlings	5.10	17.80	
Peas	10.00	20.00	20.00
Faba bean		5.00	10.00
Rapeseed oil		1.50	
Sunflower meal	2.00		
Rapeseed meal	1.10	7.00	5.00
Soybean meal	8.44		
L-lysine HCl	0.33	0.26	0.25
DL-methionine	0.04	0.05	0.09
L-threonine	0.09	0.09	0.10
L-tryptophan	0.01	0.03	0.03
Sodium chloride	0.45	0.45	0.45
Monocalcium	0.19		0.01
Calcium carbonate	1.05	1.10	1.12
Trace elements and mineral premix^*b*^	0.50	0.50	0.50
Phytase G5000	0.02	0.01	0.01
**Chemical composition, g/kg**			
Dry matter^*c*^	886	884	885
Organic matter^*d*^	838	833	839
Crude protein^*c*^	148	151	147
Crude fat^*c*^	21.3	40.5	18.4
Crude fiber^*c*^	31.2	41.3	34.3
Ca^*d*^	6.67	6.74	6.67
P^*d*^	4.35	4.67	3.86
P digestible^*d*^	2.35	2.33	2.36
Na^*d*^	1.75	1.74	1.75
K^*d*^	6.62	6.862	6.17
GE, MJ/kg^*c*^	15.89	16.34	15.84
NE, MJ/kg^*d*^	9.82	9.82	9.83
**Environmental impacts of diets, per kg of feed**^*e*^			
CC (g CO_2_-eq)	518	378	338
NRE (MJ)	5.13	4.58	3.11
AC (molc H^+^-eq)	0.0093	0.0082	0.0075
EU (g ${\text{PO}}_{4}^{3-}$-eq)	4.08	3.50	3.95
LO (m^2^year)	1.43	1.39	1.61
PD (g P)	4.09	2.53	2.83

a *Diet fed in pellet form*.

b *Provided per kilogram of complete diet: vitamin A, 1,000,000 IU; vitamin D, 3,200,000 IU; vitamin E, 4,000 mg; vitamin B1, 400 mg; vitamin B2, 800 mg; calcium pantothenate, 2,170 mg; niacin, 3,000 mg; vitamin B12, 4 mg; vitamin B6, 200 mg; vitamin K3, 400 mg; folic acid, 200 mg; biotin, 40 mg; choline chloride, 100,000 mg; iron (sulfate), 11,200 mg; iron (carbonate), 4,800 mg; copper (sulfate), 2,000 mg; zinc (oxide), 20,000 mg; manganese (oxide), 8,000 mg; iodine (iodate), 40 mg; cobalt (carbonate), 20 mg; and selenium (selenite), 30 mg*.

c *Analyzed values*.

d *Calculated values*.

e *CC, climate change; NRE, non-renewable and fossil energy demand; AC, acidification; EU, eutrophication; LO, land occupation; PD, P demand*.

**Table 2:** Composition of experimental finishing diets^*a*^.

**Diets**	**Control-diet**	**Eco-diet**	**Local-diet**
**Ingredients, %**			
Corn	25.20	37.40	2.45
Wheat	30.20		21.70
Triticale	10.00	14.60	10.00
Barley	7.00		34.50
Wheat middlings	5.00	19.50	
Peas	10.00	26.04	27.48
Faba bean			1.40
Sugar beet pulp	2.60		
Sunflower meal	2.00		
Rapeseed meal	1.00		
Soybean meal	4.60		
L-lysine HCl	0.31	0.22	0.22
DL-methionine	0.03	0.06	0.08
L-threonine	0.08	0.08	0.09
L-tryptophan	0.01	0.04	0.03
Sodium chloride	0.45	0.45	0.45
Monocalcium	0.11		0.05
Calcium carbonate	0.90	1.10	1.05
Trace elements and mineral premix^*b*^	0.50	0.50	0.50
Phytase G5000	0.01	0.01	0.01
**Chemical composition, g/kg**			
Dry matter^*c*^	887	880	884
Organic matter^*d*^	843	834	840
Crude protein^*c*^	132	136	135
Crude fat^*c*^	22.2	27.9	17.1
Crude fiber^*c*^	34.1	34.8	33.9
Ca^*d*^	6.16	6.20	6.11
P^*d*^	3.96	4.22	3.55
P digestible^*d*^	2.14	2.14	2.14
Na^*d*^	1.82	1.72	1.75
K^*d*^	5.93	6.59	5.93
GE, MJ/kg^*c*^	16.03	16.02	15.83
NE, MJ/kg^*d*^	9.85	9.85	9.87
**Environmental impacts of diets, per kg of feed**^*e*^			
CC (g CO_2_-eq)	479	364	339
NRE (MJ)	5.06	4.55	3.06
AC (molc H^+^-eq)	0.0094	0.0077	0.0074
EU (g ${\text{PO}}_{4}^{3-}$-eq)	3.98	3.60	4.06
LO (m^2^year)	1.41	1.40	1.68
PD (g P)	3.37	2.22	2.87

a *Diet fed in pellet form*.

b *Provided per kilogram of complete diet: vitamin A, 1,000,000 IU; vitamin D, 3,200,000 IU; vitamin E, 4,000 mg; vitamin B1, 400 mg; vitamin B2, 800 mg; calcium pantothenate, 2,170 mg; niacin, 3,000 mg; vitamin B12, 4 mg; vitamin B6, 200 mg; vitamin K3, 400 mg; folic acid, 200 mg; biotin, 40 mg; choline chloride, 100,000 mg; iron (sulfate), 11,200 mg; iron (carbonate), 4,800 mg; copper (sulfate), 2,000 mg; zinc (oxide), 20,000 mg; manganese (oxide), 8,000 mg; iodine (iodate), 40 mg; cobalt (carbonate), 20 mg; and selenium (selenite), 30 mg*.

c *Analyzed values*.

d *Calculated values*.

e *CC, climate change; NRE, nonrenewable and fossil energy demand; AC, acidification; EU, eutrophication; LO, land occupation; PD, P demand*.

### Animal Study

The experiment was conducted in accordance with French legislation on animal experimentation and approved by the Regional Ethics Committee (authorization: 2019041815163846).

A total of 96 Pietrain × (Large White × Landrace) pigs were raised in a single experimental room; each pig was weighed and fed individually using an automatic weighing and feeding system. The experiment was conducted at the INRAE Pig Physiology and Phenotyping Experimental Facility (UE3P) located in Saint Gilles, France (https://doi.org/10.15454/1.5573932732039927E12). Pigs were distributed among three experimental groups: Control-diet, Eco-diet, and Local-diet (Tables 1, 2). Pigs were assigned to the experimental treatments according to sex and litter origin according to a randomized complete block design. Therefore, each experimental group had an equal number of entire males and females (*n* = 16 per group per sex). Pigs in the experiment started at 40 kg average BW and ended at 115 kg average BW. Based on BW, pigs received experimental diets that met the requirements for growing (40–65 kg) or finishing (65–115 kg). Prior to entering the experimental room, pigs were tagged in the right ear with a serial number and an RFID chip for identification in the sorter (which also served as the weighing machine) and at the automated feeders. A detailed description of the feeding system used in this experiment was provided by Pomar et al. (23). The experimental room had two feeding zones that the pigs accessed by passing through an automatic sorter. Each feeding zone was equipped with four automatic feeders. The sorter was programmed in random order so pigs could access either zone at random. Feed and water were provided *ad libitum*. Six pigs were removed from the experiment as a result of bodily injuries (4) or death (2) from causes unrelated to the experimental diets.

Live weight was measured automatically when the pigs passed through the automatic sorter. For each pig, average daily BW was calculated as the average of all of the BW recordings taken each day. Individual daily feed intake was calculated based on the recordings of the automatic feeding system according to the number of feed servings (in theory, one serving = 25 g) and a calibration factor. Calibration measurements were performed weekly on all feeders to adjust for the actual amount of feed delivered per serving. From these measurements, the calibration factor was calculated as the ratio of the actual amount delivered to the theoretical value.

All pigs fasted 24 h before slaughter; BW at slaughter was the final measurement taken as the pigs passed through the automatic sorter upon their departure for the slaughterhouse. Carcass characteristics, including carcass weight, lean meat percentage, and carcass yield, were measured at the slaughterhouse.

### Life Cycle Assessment

#### Goal and Definition of Scope

**Figure 1:**
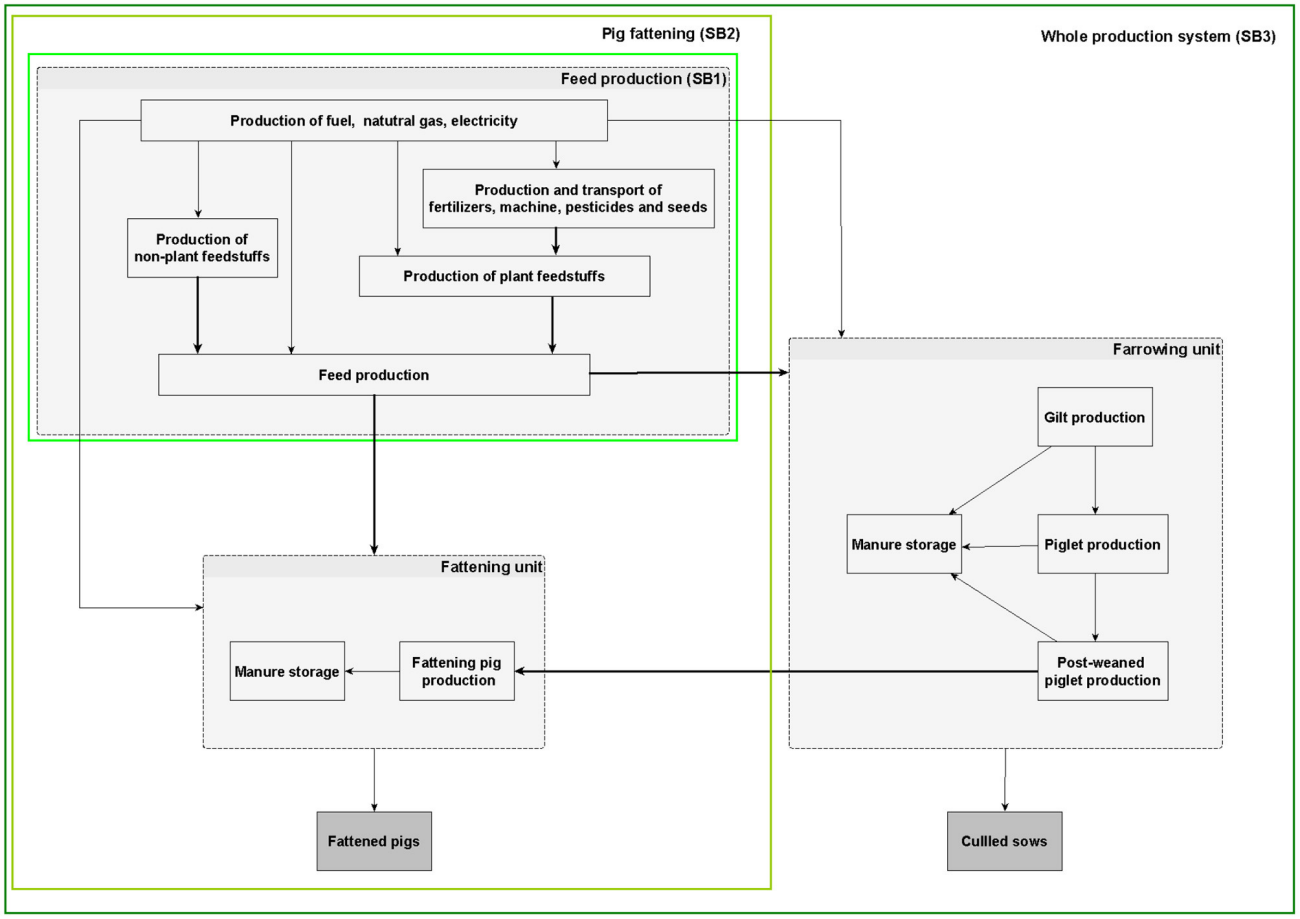
Description of the three system boundaries (SB) considered in this study.

The potential environmental impacts were calculated for each of the three experimental treatments using LCA; this approach evaluated the whole process of pig production, farrow to finish, in this case as carried out in Brittany, northwest France. The pig production system considered was a conventional growing–finishing pig farm in which animals are raised indoors on a slatted floor and manure is collected and stored externally as liquid slurry in an uncovered pit. To investigate the specific mechanisms by which each of the feed strategies modified the environmental impacts of pig production, three different system boundaries were considered (Figure 1):

- For the feed production process, the system boundaries (SB1) included the production and transport of feed ingredients and feed production, either at the feed factory (for the Control-diet and Eco-diet) or on-farm (Local-diet). In this case, the functional unit considered was 1 kg of feed leaving the feed factory (or, for the Local-diet, leaving the farm feed unit).
- For the fattening process, the system boundaries (SB2) were derived from Monteiro et al. (24) and included the production and transport of feed ingredients to the feed factory, the production process for growing and finishing feeds, transport of the feed to the farm (for the Control-diet and Eco-diet), growing to finishing pig production, and manure storage. For these system boundaries, the functional unit was 1 kg of live weight gain during fattening.
- For the entire farrow-to-finish production process, the system boundaries (SB3) were derived from Dourmad et al. (5) and included the production of piglets (farrowing unit) as well as the postweaning and growing–finishing periods, the production and transport of feed ingredients to the feed factory, the production of feed on-farm or at the feed factory, and emissions from animals and manure storage. The associated functional unit was 1 kg of live weight at the farm gate, including fattening pigs and culled sows.

For the fattening and farrow-to-finish analyses, the environmental impacts were calculated individually for each pig according to its individual performance. To this end, the impacts associated with piglet production and the postweaning period were accounted for in each pig using the following equation:

(5) $$\begin{array}{l} Impact_{ij}^{SB3}=\\ \;\frac{Impact_{ij}^{Fattening}\times NbW+\left(Impact_{i}^{PW}\times NbP\right)+Impact_{i}^{FU}}{LW_{j}^{Slaughter}\times NbS+\;\left(LW^{CulledSow}-LW^{Gilt}\right)\times CullingRate} \end{array}$$

with $Impact_{ij}^{SB3}$: the impact i of pig j per kilogram of live weight at the farm gate, NbW: the number of weaners produced per sow per year, NbP: the number of weaned piglets per sow per year, NbS: the number of slaughtered pigs per sow per year, $Impact_{ij}^{Fattening}$: the total impact i of fattening pig j during the fattening period, $Impact_{i}^{PW}$: the total impact i of one pig during the postweaning period, $Impact_{i}^{FU}$: the total impact i of one sow over 1 year, $LW_{j}^{Slaughter}$: the live weight of pig j at the farm gate, *LW*^*CulledSow*^: the live weight of the culled sow at the farm gate, *LW*^*Gilt*^: the live weight of the gilt at first mating, and *CullingRate*: the replacement rate of sows on the farm.

#### Life Cycle Inventories

The environmental impacts of the feed ingredients that were incorporated in the growing and finishing feeds came from the ECOALIM dataset (version 7, October 1, 2019, https://www6.inrae.fr/ecoalim/) (6). Hypothesized impacts of the transport of feed ingredients from field to feed factory and of the transport of feeds from feed factory to pig farm came from Méda et al. (25). The impacts of feeds for sows and postweaning piglets came from Méda et al. (25). Estimates of energy consumption in buildings were obtained from Dourmad et al. (5) for sows, postweaning piglets, and fattening pigs. The impact of processing in the feed factory was included in the life cycle inventories of Control-diet and Eco-diet feeds, with the assumptions that grinding and pelleting required 41 kWh of electricity and 20.5 kWh of natural gas per ton of feed produced (26). For on-farm feed production, grinding and mixing were estimated to require 18 kWh of electricity per ton of feed produced (27). The construction of buildings and manure storage units, as well as veterinary and cleaning products, were not included in the life cycle inventories. Background data for energy and transport came from ecoinvent v3.5 (28) included in the Agribalyse v3 database available in SimaPro®.

Nitrogen (N), phosphorus (P), and potassium excretions of sows, postweaning piglets, and fattening pigs were calculated using the mass balance approach of BRSPorc (29). Excretion of total ammoniacal N was calculated for fattening pigs as urinary N, resulting from the difference between the intake of digestible N and its retention in the body. For sows and piglets, excretion of ammoniacal N was calculated as a fixed proportion of N excretion, established by expert knowledge (Sandrine Espagnol, personal communication). Gaseous losses of nitrogen from manure in buildings and during manure storage were calculated in one of two ways: for NH_3_, NO_x_, and N_2_ emissions, conversion factors from the European Monitoring and Evaluation Programme (EMEP) (2016) emission guidebook were applied to excreted ammoniacal N, and for N_2_O, conversion factors were applied to total N excreted as per IPCC (2006). Excretion of organic matter was determined as a function of feed composition; emissions of CH4 from enteric fermentation and from manure storage were calculated using methods from the Intergovernmental Panel on Climate Change (IPCC) (2006) and Rigolot et al. (30, 31).

#### Life Cycle Impact Assessment

Six categories of impacts were calculated: CC, NRE, AC, EU, LO, and PD. The indicator result for each category was determined by multiplying the aggregated resources used and the aggregated emissions of each individual substance by a characterization factor unique to each applicable category. For CC (kg CO_2_-eq) and AC (molc H^+^-eq), impacts were estimated according to the International Reference Life Cycle Data (ILCD) System (32). EU (kg ${\text{PO}}_{4}^{3-}$-eq) and LO (m^2^year) were calculated using the approach of the CML, and NRE (MJ) was predicted according to CED v1.08 (implemented in SimaPro® v. 8.0.5.13). All calculations were made with a publicly available software developed in Python 3.7 (https://doi.org/10.15454/PIJXCR) and extracted from the model developed by Cadéro et al. (33), which contains all equations and inputs for LCA described in this manuscript.

### Statistical Analysis

Since all pigs were raised in a single experimental room, the statistical unit was the pig. Animal performance and farm-gate environmental impacts were subjected to an analysis of variance that tested the effects of gender (G), sire (S), and experimental diet (D) while taking into account the random effect of sire; the pig was the statistical unit considered. For this, the LME (linear mixed-effects) function from the NLME package of R software [version 3.5.1, (34)] was used, and results were considered significant for *p*-values lower than 0.05.

## Results

### Experimental Diets

#### Diet Composition

The mean composition of each experimental diet with respect to ingredient (%) and nutritional content (g/kg) is provided in Tables 1, 2. Compared with the Control-diet, the MO formulations contained a smaller proportion of cereals and oil meals and a larger proportion of protein-rich crops and coproducts. Specifically, the Control-diet contained an average of 71% cereals, 10% protein-rich crops (peas), 9.5% oil meals, 5% wheat middlings, 2.6% sugar beet pulp (only finishing), and 2.7% (growing) or 2.4% (finishing) additives (amino acids, vitamins, trace elements, and phytase). For the Eco-diet, the growing feed contained 46.2% cereals, 25% protein-rich crops (peas and faba beans), 17.8% wheat middlings, 7% rapeseed meal, 1.5% rapeseed oil, and 2.5% additives (amino acids, vitamins, trace elements, and phytase), while the finishing feed contained 52% cereals, 26% protein-rich crops (peas and faba beans), 19.5% wheat middlings, and 2.5% additives (amino acids, vitamins, trace elements, and phytase). The Local-diet contained 62.5% (growing) or 68.6% (finishing) cereals, 30% (growing) or 28.9% (finishing) protein-rich crops, 5% oil meals (only growing), and 2.5% additives (amino acids, vitamins, trace elements, and phytase).

#### LCA Impacts of the Diets (Per Kilogram of Feed)

The detailed LCA impacts of the experimental growing and finishing diets (expressed per kilogram of feed) are provided in Tables 1, 2. Compared with the Control-diet, the growing and finishing Eco-diets reduced the impact of CC by 27.0 and 24.0%, NRE by 10.8 and 9.9%, AC by 11.8 and 18.2%, EU by 14.2 and 9.6%, LO by 3.4 and 0.5%, and PD by 38.1 and 34.1%, respectively. Again compared with controls, the growing and finishing Local-diets reduced the impact of CC by 34.7 and 29.2%, NRE by 39.4 and 39.5%, AC by 20.1 and 21.6%, EU by 3.2% (only for the growing diet), and PD by 30.8 and 34.1%, respectively. However, the impact of EU increased by 2.0% with the finishing Local-diet, and the impact of LO increased with both Local-diets: 12.2% with the growing diet and 19.6% with the finishing diet. When we compared the Eco-diet and Local-diet, we found very similar patterns regardless of the growth stage targeted: the Eco-diet had higher CC impacts (10.6% for growing and 6.9% for finishing), higher NRE impacts (32.1 and 32.8%, respectively), higher AC impacts (9.5 and 4.2%, respectively), lower EU impacts (12.9 and 12.8%, respectively), lower LO impacts (16.1 and 20.2%, respectively), and lower PD impacts (11.9 and 29.3% respectively) than the Local-diet.

### Animal Performance

Indicators of pig performance are presented in Table 3. Measurements of initial, growing, and final BW did not differ between the experimental groups (40.7, 61.0, and 113.0 kg on average; *p* = 0.915, *p* = 0.852, and *p* = 0.943, respectively). During the growing period, average daily gain (ADG) (883 g/d), average daily feed intake (ADFI) (2.05 kg/d), and total water consumption per pig per day (4.59 L) were similar among the experimental groups (*p* = 0.336, *p* = 0.442, and *p* = 0.486, respectively). Pigs fed the Local-diet had the highest feed conversion ratio (FCR), while those fed the Eco-diet had the lowest (2.48 vs. 2.24 kg/kg; *p* < 0.01); the FCR for the Control-diet group was intermediate in value (2.32 kg/kg). During the finishing period, we did not observe any significant differences between the experimental groups with respect to ADG (947 g/d), ADFI (2.75 kg/d), FCR (2.69 kg/kg), and daily water consumption per pig (5.59 L) (*p* = 0.505, *p* = 0.108, *p* = 0.569, and *p* = 0.195, respectively).

**Table 3:** Effect of diets on the growth performance of pigs.

	**Control-diet**	**Eco-diet**	**Local-diet**	**RSD**	**Statistics**
Animals, *n*	31	29	30		
Initial BW, kg	40.8	40.5	40.9	0.11	
Growing BW, kg	61.4	61.1	60.6	0.09	
Final BW, kg	113	113	113	0.08	G^**^
**Growing period**			
Initial BW, kg	40.8	40.5	40.9	0.10	
Growing BW, kg	61.4	61.1	60.6	0.09	
Duration, d	23	23	23		
Total feed intake, kg/pig	47.3	45.9	48.0	0.15	
ADG, g/d	896	898	854	0.15	G^**^
ADFI, kg/pig/d	2.06	1.99	2.09	0.15	G^***^
FCR	2.32^ab^	2.24^b^	2.48^a^	0.11	G^*^, S^**^,D^**^
Daily water consumption, L/pig/d	4.47	4.47	4.84	0.31	G^*^, S^*^
**Finishing period**			
Initial BW, kg	61.4	61.1	60.6	0.09	
Final BW, kg	113	113	113	0.08	G^**^
Duration, d	55	55	55		
Total feed intake, kg/pig	142.5	144.3	149.9	0.11	
ADG, g/d	938	940	963	0.10	G^**^
ADFI, kg/pig/d	2.69	2.72	2.83	0.11	S^***^
FCR	2.65	2.69	2.72	0.09	G^***^,S^**^
Daily water consumption, L/pig/d	5.26	5.52	5.99	0.31	S^***^
**Growing–finishing period**			
Duration, d	78	78	78		
Total feed intake, kg/pig	189.8	190.2	198.0	0.11	
ADG, g/d	926	927	931	0.10	G^***^
ADFI, kg/pig/d	2.50	2.50	2.60	0.11	S^***^
FCR	2.64	2.64	2.74	0.11	G^***^, S^***^
Daily water consumption, L/pig/d	5.02	5.20	5.64	0.30	S^**^
Carcass yield, %	78.2	78.3	78.4	0.01	G^**^, S^*^
Lean meat, %	61.0	61.3	60.7	0.03	G^*^
Carcass weight, kg	88.4	88.3	89.0	0.08	G^*^

*ADG, average daily gain (g/d); ADFI, average daily feed intake (kg/pig/d); FCR, feed conversion ratio (ADFI/ADG); G, gender; S, sire; RSD, Residual standard deviation*;

* *p < 0.05*,

** *p < 0.01*,

*** *p < 0.001*.

a,b *Means with different superscripts (a, b) are significantly different between the experimental diet (p < 0.05)*.

When we examined the performance of pigs over the total experimental period, we detected no significant differences in ADG (928 g/d), ADFI (2.53 kg/d), FCR (2.67 kg/kg), and total water consumption per pig per day (5.29 L) among the three experimental groups (*p* = 0.976, *p* = 0.188, *p* = 0.139, and *p* = 0.238, respectively). Values of carcass yield (78.3%), lean meat percentage (61%), and carcass weight (88.6 kg) were also similar in the three groups (*p* = 0.819, *p* = 0.362, and *p* = 0.919, respectively).

Globally, we observed significant (*p* < 0.01) differences between females and entire males with respect to final BW (110.4 vs. 116.0 kg), ADG (895 vs. 964 g/d), FCR (2.76 vs. 2.57 g/g), carcass yield (78.59 vs. 78.02%), lean meat percentage (60.6 vs. 61.4%), and carcass weight (86.8 vs. 90.5 kg).

### Environmental Impacts at Farm Gate

#### LCA Impacts of Pig per Kilogram of BW Gain

The environmental impacts of growing–finishing pigs are reported in Table 4, per kilogram of BW gain (BWG). Compared with the Control-diet, the Eco-diet significantly reduced CC by 15.0%, NRE by 9.2%, AC by 7.5%, EU by 7.9%, and PD by 35.3% (*p* < 0.01). The LO impact was similar between the Control-diet and the Eco-diet (4.65 and 4.58 m^2^year, respectively; *p* = 0.808). The Local-diet, in comparison with the Control-diet, significantly reduced CC by 18.6%, NRE by 32.7%, and PD by 15.5% (*p* < 0.01). No modification of AC (0.112 and 0.104 molc H^+^-eq, respectively) and EU (226 and 208 g ${\text{PO}}_{4}^{3-}$-eq, respectively) impacts was observed between the Control-diet and the Local-diet (*p* = 0.316 and *p* = 0.221, respectively). However, relatively to the Control-diet, the Local-diet significantly increased LO by 22.1% (*p* < 0.01). The Eco-diet and the Local-diet had similar CC impact (2.04 and 1.95 kg CO_2_-eq, respectively; *p* = 0.180). The Eco-diet had higher NRE impact than the Local-diet (increased by 25.9%), but lower AC (decreased by 4.6%), EU (decreased by 12.6%), LO (decreased by 24.0%), and PD (decreased by 30.7%) impacts than the Local-diet (*p* < 0.01).

**Table 4:** Environmental impacts at farm gate (per kilogram of body weight gain in fattening unit and per kilogram of pig live weight at farrow-to-finish farm gate).

**Impacts at fattening unit gate (per kilogram of body weight gain)**					
	**Control-diet**	**Eco-diet**	**Local-diet**	**RSD**	**Statistics**
CC (kg CO_2_-eq)	2.40^a^	2.04^b^	1.95^b^	0.14	G^**^, S^***^, D^***^
NRE (MJ)	19.06^a^	17.30^b^	12.82^c^	0.19	G^***^, S^***^, D^***^
AC (molc H^+^-eq)	0.112^a^	0.104^b^	0.108^a^	0.11	G^**^, S^***^, D^**^
EU (g ${\text{PO}}_{4}^{3-}$-eq)	226^a^	208^b^	234^a^	0.11	G^**^, S^***^, D^***^
LO (m^2^year)	4.65^b^	4.58^b^	5.68^a^	0.14	G^***^, S^***^, D^***^
PD (g P)	115^a^	74^c^	97^b^	0.20	G*, S^***^, D^***^
**Impacts at farm gate (per kilogram of body weight)**					
CC (kg CO_2_-eq)	2.40^a^	2.17^b^	2.11^b^	0.08	G^***^, S^***^, D^***^
NRE (MJ)	22.37^a^	21.22^b^	18.30^c^	0.10	G^***^, S^**^, D^***^
AC (molc H^+^-eq)	0.085^a^	0.080^b^	0.083^a^	0.08	G*, S^***^, D^**^
EU (g ${\text{PO}}_{4}^{3-}$-eq)	198^a^	186^b^	203^a^	0.08	G^**^, S^***^, D^***^
LO (m^2^year)	4.33^b^	4.28^b^	4.99^a^	0.1	G^**^, S^***^, D^***^
PD (g P)	122^a^	95^c^	110^b^	0.12	G^***^, S^***^, D^***^

*CC, climate change; NRE, non-renewable and fossil energy demand; AC, acidification; EU, eutrophication; LO, land occupation; PD, P demand; G, gender; S, sire; D, diet; RSD, residual standard deviation*;

** *p < 0.01*,

*** *p < 0.001*;

a,b,c *Means with different superscripts (a, b, c) are significantly different between the experimental diet (p < 0.05)*.

#### LCA Impacts of Pig per Kilogram of BW at Farm Gate

The details of LCA impacts of pig production at farm gate per kilogram of BW are also presented in Table 4. In comparison with the Control-diet, the Eco-diet significantly decreased the CC impact by 9.7%, the NRE impact by 5.1%, the AC impact by 6.2%, the EU impact by 5.8%, and the PD impact by 21.9% (*p* < 0.01). No difference in LO impact was observed between the Control-diet and the Eco-diet (4.33 and 4.28 m^2^year, respectively; *p* = 0.808). The Local-diet significantly decreased CC impact by 12.2%, NRE impact by 18.2%, and PD impact by 9.8% in comparison with the Control-diet (*p* < 0.01). No modification of AC (0.085 and 0.080 molc H^+^-eq, respectively) and EU (198 and 186 g ${\text{PO}}_{4}^{3-}$-eq, respectively) impacts was observed between the Control-diet and the Local-diet (*p* = 0.227 and *p* = 0.174, respectively). However, relatively to the Control-diet, the Local-diet significantly increased the LO impact by 15.4% (*p* < 0.01). The CC impact per kilogram of BW was similar between the Eco-diet and the Local-diet (2.17 and 2.11 kg CO_2_-eq, respectively; *p* = 0.129). In comparison with the Local-diet, the Eco-diet had higher NRE impact (increased by 13.8%) and lower AC (decreased by 3.6%), EU (decreased by 9.0%), LO (decreased by 16.5%), and PD (decreased by 15.4%) impacts (*p* < 0.01).

## Discussion

### Effectiveness of MO Formulation Approach on the Environmental Impacts of Feeds

In the least-cost formulated Control-diet, the main ingredients were cereals (71%), supplemented with protein-rich crops (10%), oil meals (9.5%), and coproducts of wheat (5%) (Table 1). The feeds obtained with the MO formulation approach differed from this in important ways: the Eco-diet was characterized by a lower proportion of cereals, while both the Eco-diet and Local-diet contained higher proportions of alternative protein sources and smaller proportions of oil meals, especially soybean meal, which was substituted with rapeseed meal, peas, or faba beans (Tables 1, 2). These changes were consistent with those reported from previous efforts to include environmental objectives in the calculation of feed formulations (9, 10). Here, the composition of the diets obtained with the MO formulation was close to that formulated by Garcia-Launay et al. (10). Similarly, Mackenzie et al. (9) reported that diets formulated with environmental objectives in mind included a smaller proportion of cereals and a higher proportion of coproducts than diets formulated with economic objectives only (6).

With MO formulations, the relative incorporation rates of feed ingredients are shaped by trade-offs between the nutritional value, cost, and environmental impacts of each ingredient. Compared with cereals, protein-rich ingredients obtained from legume seeds, like peas and faba beans, are characterized by lower CC and NRE impacts because, unlike cereals, they do not require mineral nitrogen fertilization (6); this was the reason for their relatively high contributions to the Eco-diet and Local-diet. However, because of lower production yields, locally produced protein-rich crops have a higher LO impact than cereals (6). The production and utilization of crops on farm—and thus a reduced reliance on transport—decreased the average CC impact of ingredients by 8%, NRE by 14%, AC by 4.5%, and EU, LO, and PD by 2.5%. The environmental impacts of coproducts were relatively low, partly due to the economic allocation of impacts. Moreover, the industrial processes associated with their production are not input intensive (6). For example, the CC impact of wheat middlings was 75% lower than that of wheat, even though its crude protein content is about 50% higher; similar patterns were observed for other impacts as well. Among all the feed ingredients used in this study, wheat middlings had the lowest value for all impact categories (except for the AC impact, for which it was the second lowest). Similarly, the LCA impacts of rapeseed meal are about 60% lower than those of rapeseed grain. On the other end of the spectrum is soybean meal, which is associated with serious environmental impacts: most soybean meal in France is imported from South America, where agriculture-associated deforestation remains widespread (35). This means that soybean meal has a CC impact four times higher and an NRE impact three times higher than rapeseed meal, while its price and protein content are only about 30–40% higher.

In the MO formulation approach, the objective function weighted the environmental impacts of CC more heavily (× 2) than those of NRE, LO, and PD (× 1) because the mitigation of CC is considered to be a priority [Paris Agreement, 2015; (10)]. This was the underlying reason for the higher proportions of protein-rich crops and wheat coproducts and the reduced proportions of cereals and imported soybean meal in diets formulated with both environmental and economic objectives. Specifying weighting factors to the various environmental impacts is still a matter of debate in the literature. As recommended by Garcia-Launay et al. (10), in this study, we chose a pragmatic approach that consists in providing the same factors to all global impacts (NRE, PD, and LO) and a higher factor to CC. Performing feed formulation while accounting for various environmental impacts requests weighting the various impacts in the objective function. Indeed, formulating while minimizing a single impact leads to pollution transfer to other impacts or the increased use of limited resources (9). Using constraints on the various environmental impacts requires a step-by-step approach to find the adequate constraints for each single impact. Other approaches include basing weighting factors on monetary valuation, public opinion, or the state of the receiving environment (36). Although relevant for comparing the LCA of various scenarios, using these approaches for optimization may increase greatly impacts that are associated with lowest weighting factors.

When we compared the MO-formulated feeds to the least-cost Control-diet, we found that the environmental impacts of the Eco-diet were universally smaller. The Local-diet, instead, had smaller impacts with respect to CC, NRE, AC, and PD; no change for EU; and an increased impact on LO. In the least-cost formulation, imported soybean meal accounted for 19.5% of the CC impact and 12.9% of the NRE impact. Our results are consistent with those of Eriksson et al. (7) and van Zanten et al. (8), who reported a similar reduction in environmental impacts after replacing soybean meal with peas or rapeseed meal. Specifically, Eriksson et al. (7) substituted soybean meal with peas and rapeseed meal in growing–finishing pig diets and observed a reduction of 7% in the CC impact and 10% in the NRE impact; van Zanten et al. (8) showed that replacing soybean meal with rapeseed meal in a finishing pig diet reduced the CC impact by 10%. Here, the AC impact of the least-cost formulation (0.0094 molc H^+^-eq) was a little higher than that of the Eco-diet (0.0078 molc H^+^-eq) and the Local-diet (0.0074 molc H^+^-eq) (Supplementary Table 6). This was the result of higher proportions of high-AC cereals in the least-cost diet and a shift to higher proportions of low-AC protein-rich crops and wheat coproducts in the MO formulations. In addition, a higher proportion of wheat middlings in the Eco-diet resulted in a reduced EU impact (3.6 g ${\text{PO}}_{4}^{3-}$-eq) relative to that of the least-cost formulation (4.0 g ${\text{PO}}_{4}^{3-}$-eq) and the Local-diet (4.0 g ${\text{PO}}_{4}^{3-}$-eq) (Supplementary Table 6). However, the reductions in AC and EU impacts with the MO formulation were minor compared with those observed for other impacts, mainly due to the fact that the objective function did not include either AC or EU. Patterns of PD among the diets (3.5 g P with least-cost formulation compared to 2.3 and 2.9 g P with Eco-diet and Local-diet, respectively) (Supplementary Table 6) could also be traced back in large part to the Control-diet's reliance on soybean meal, which has a P demand three times higher than that of other ingredients; the inclusion of wheat middlings in the Eco-diet also played a role, as the P demand of wheat middlings is five times lower than that of other ingredients. Overall, then, the MO formulation approach appeared to be quite effective in reducing the environmental impacts of pig fed. However, in one case, the MO approach significantly increased one environmental impact: the Local-diet had an LO impact that was about 18% higher (1.66 m^2^year) than that of the least-cost formulation (1.41 m^2^year) or the Eco-diet (1.40 m^2^year) (Supplementary Table 6). Because of their generally lower yields, protein-rich crops need more land than cereals or coproducts to produce the same quantities (6–8). Moreover, for some crops, like soybean meal grown in South America, more than one crop can be harvested per year, which results in reduced values for LO. The Local-diet contained a similar proportion of cereals as the Control-diet but a higher proportion of protein-rich crops, and these two ingredient families have strong impacts on LO.

In agreement with the literature, we confirm that substituting cereals and soybean meal with alternative protein sources (rapeseed meal, peas, faba beans, or wheat middlings) is an efficient means of reducing the environmental impacts of pig feed; the overall balance of impacts can be further mediated by factors associated with ingredient production (i.e., locally produced vs. imported). The incorporation of ingredients that are produced locally further decreases the CC and NRE impacts of feed because of a reduction in transportation requirements; however, this strategy also increases the impact of LO because it relies more heavily on lower-yielding crops.

### Animal Performance

Previous studies that have included environmental objectives in the calculation of feed formulations (9, 11) based their models of environmental impacts on the assumption that animal performance would be unaffected by feed composition. However, feeding strategies that minimize environmental impacts typically contain higher proportions of protein-rich crops and coproducts that may vary in their nutritional composition and energy, fiber, or protein content (12, 13). Such variability may have consequences on feed intake or digestibility, with potential repercussions for animal performance (14, 15). With an experimental approach, Shaw et al. (14) showed that the incorporation of wheat middlings in pig feed had a negative effect on growth. Similarly, the substitution of soybean meal with rapeseed meal may also decrease pig performance (15). Therefore, one potential concern with MO-formulated diets is that the environmental improvements obtained for the feed might be offset, partially or in total, by losses in pig performance. Here, we observed a slightly lower ADG and a slightly higher ADFI in the Local-diet group during the growing period, which resulted in an FCR that was significantly higher than that of the two other groups. We do not fully understand this difference in animal performance, as the estimated net energy and lysine concentrations in the three diets were formulated to be equal and based on the real nutritional values of ingredients (dry matter, organic matter, crude protein, and gross energy). This response might be due to interactions among ingredients that then affect digestibility, but in any event, it deserves further study. During the finishing period, animal performance was unaffected by the feeding strategies. The three groups all demonstrated similar carcass characteristics, including carcass weight, lean meat percentage, and carcass yield, resulting in similar carcass value. Over the fattening period as a whole, the MO formulations had no effect on animal performance, indicating that this innovative formulation method is effective in reducing environmental impacts without compromising performance or carcass quality.

Overall, unlike previous studies, we found no evidence of impaired performance due to the inclusion of alternative protein sources (rapeseed meal, protein-rich crops, or cereal coproducts) in animal diets. This difference may be related to the relatively higher levels of variability in the nutritional value of these ingredients. Instead of designing diets based on published average nutritional values, our study analyzed the real nutritional value of ingredients to determine the composition of diets. In addition, diets were carefully formulated to ensure they met the minimum nutritional content for pigs, in order to meet the requirements for net energy and standardized amino acid content established by the performance objective.

### Environmental Impacts of Fattening Unit and Farrow-To-Finish Production

**Figure 2:**
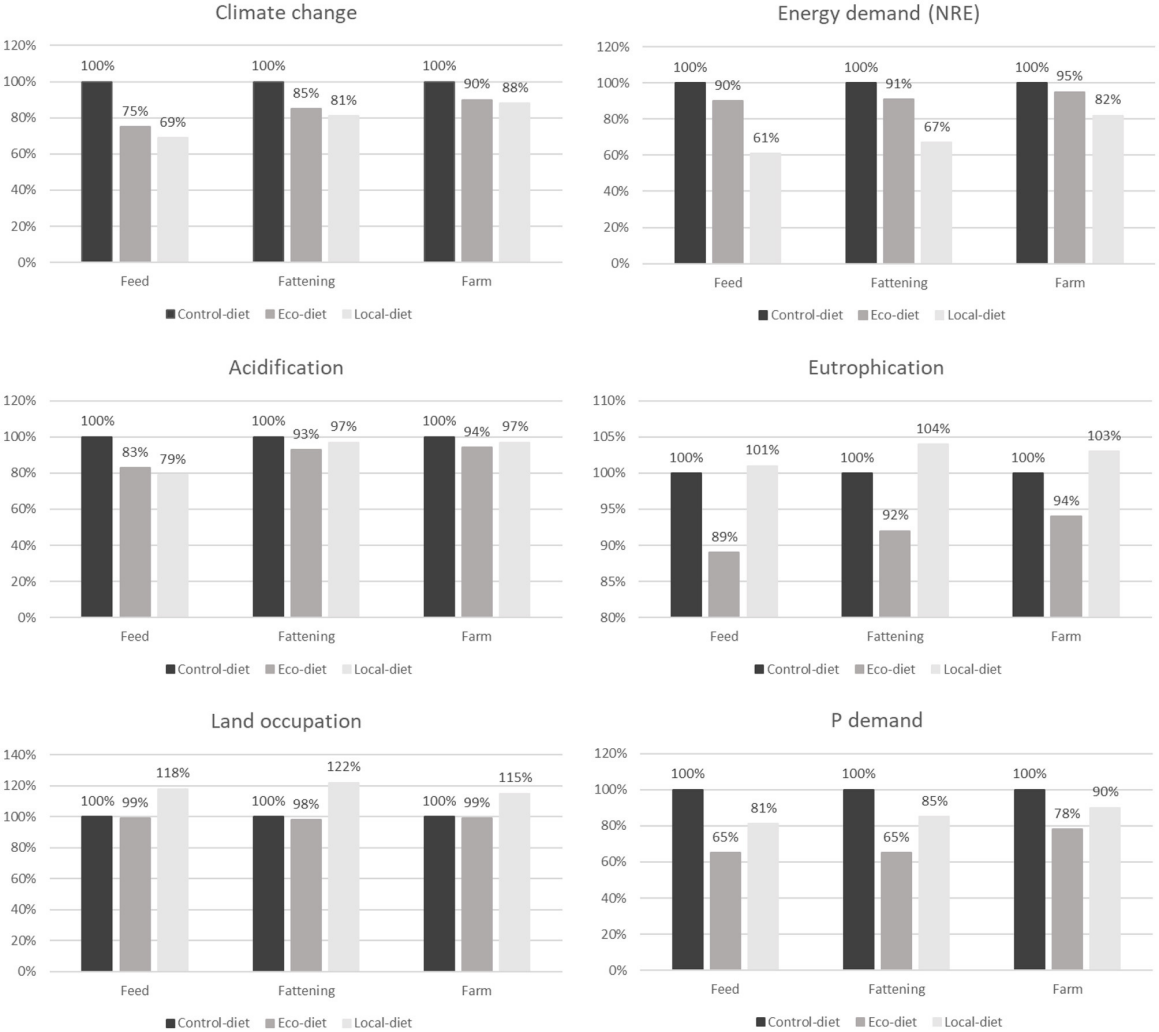
Effect of the strategy for diet formulation on environmental impacts of the average feed and of pig production at fattening unit gate or at farm gate. Values are expressed as a percentage of the value obtained for the Control-diet strategy.

In our study, we calculated the environmental impacts of each diet strategy in three contexts: with respect to the feed only (i.e., impacts arising from feed ingredients and feed production processes), in the context of a fattening unit (i.e., the cumulative impacts required to raise an animal that is ready to be transported to the slaughterhouse), and in the context of an entire farrow-to-finish production farm (i.e., the cumulative impacts related to breeding, growing, and finishing). Since animal performance was similar among the three feeding strategies tested, the effects of the different feed formulations on the environmental impacts of fattening units followed the same general pattern as those obtained for the feed only (Figure 2). In comparison with the least-cost formulation, the Eco-diet significantly reduced all impacts except LO. The extent of the reduction was the same for the feed and the fattening unit with respect to NRE (−10%), LO (−1%), and PD (−35%) (Figure 2). However, for CC, AC, and, to a lesser extent, EU, the reduction in the fattening unit was smaller in magnitude than that observed for the feed (−15 vs. −25% for CC, −8 vs. −17% for AC, and −8 vs. −11% for EU, respectively; Figure 2). This might be explained by the fact that, for NRE and LO, the feed production process made a higher relative contribution to the total impact (>90%) than it did for CC, AC, and EU (30, 50, and 60%, respectively) (24). With respect to AC and EU, the relatively small degree of improvement seen with the use of the Eco-diet compared to the Control-diet can probably be explained by the fact that emissions from housing and manure were similar between the two feeding strategies.

In the same way, the effect of the Local-diet on the overall environmental impact of a fattening unit was the same as the effect of the diet alone (relative to the Control-diet) for three categories: NRE (−39% for Local-diet compared with Control-diet), LO (+20%), and PD (+20%). Instead, the fattening-unit effect was lower in magnitude for CC, AC, and EU (−31% for feed-only vs. −20% for the fattening unit with respect to CC, −21 vs. −3% for AC, and +1 vs. +4% for EU; Figure 2). In the context of the fattening unit, then, use of the Local-diet still reduced the impacts of CC, NRE, and PD compared with the Control-diet but was not significantly different from the Control-diet with respect to the impacts on AC and EU. At the level of both feed and the fattening unit, the Local-diet significantly increased LO over control values to a similar extent (+18 and +22%, respectively).

When applied to a fattening unit, the Eco-diet was more effective in reducing the impacts of AC, EU, LO, and PD than the Local-diet; however, the impact on CC was similar between the two strategies. Furthermore, the Local-diet was more efficient in reducing the impact of NRE per kilogram of BWG (−40%) than the Eco-diet. Garcia-Launay et al. (10), Wilfart et al. (11), and Méda et al. (25) all obtained similar results based on models of animal performance.

For CC and NRE, the differences among the feeding strategies were more muted when examined in the context of a farrow-to-finish production farm than in a fattening unit, while for AC, EU, LO, and PD, the relative differences between strategies remained generally similar. Specifically, implementation of the Local-diet and Eco-diet reduced the CC impact of a production farm by only 10% compared with the Control-diet strategy (the corresponding reduction for the Local-diet and Eco-diet in fattening units being 15 and 19%, and in the feed-only analysis, 25 and 31%, respectively; Figure 2). Similarly, use of the Local-diet reduced NRE on the production farm by only 5% compared to a 39% reduction for feed only and a 33% reduction for the fattening unit. These differences are mainly related to the contributions of the farrowing and postweaning units, which consume a significant amount of energy for heating (5). Furthermore, the farrow-to-finish LCA was carried out based on the assumption that sows and piglets were given conventional (least-cost formulated) diets, and it is likely that this also contributed to the reduction (or dilution) in the apparent effects of the different fattening feeds. If MO formulations had also been applied for the phases of gestation, lactation, and weaning, it is probable that the difference between feeding strategies would have been more marked.

## Conclusion

MO formulation is a useful strategy for reducing the environmental impacts of pig production. Using this approach, we were able to select feed ingredients with lower environmental impacts, such as protein-rich crops or agricultural coproducts, and thus efficiently reduce the impacts of pig production without adverse consequences on animal performance or carcass quality. Before such diets can be applied, however, it is important to first analyze the nutritional composition of the ingredients in order to adjust the composition of the diet according to their real nutritional values. Another potential challenge could arise regarding the availability of ingredients: wide-scale incorporation of these ingredients in ecofriendly diets could result in scarcity, as protein-rich crops currently represent only 2% of cultivated land in France. Moreover, increasing demand for coproducts could affect feed prices and, consequently, the economic allocation of environmental impacts. Such potential constraints must be taken into consideration by future efforts to implement these innovative formulation methodologies at a large scale.

## Data Availability Statement

The original contributions presented in the study are publicly available. This data can be found here: https://data.inrae.fr/dataset.xhtml?persistentId=10.15454/PIJXCR10.15454/PIJXCR.

## Ethics Statement

The animal study was reviewed and approved by Regional Ethics Committee of Brittany (authorization: 2019041815163846).

## Author Contributions

FQ, LB, AW, J-YD, and FG-L contributed to the conception and design of this study. AW and FQ contributed to funding acquisition. FQ and LB were responsible for the animal experiment. FG-L provided the development of the LCA model and the following evaluation and assessment of this model. FQ wrote the first draft of the manuscript. J-YD and FG-L wrote sections of the manuscript. All authors contributed to manuscript revision and read and approved the submitted version.

## Conflict of Interest

The authors declare that the research was conducted in the absence of any commercial or financial relationships that could be construed as a potential conflict of interest.
