# Supplementary material for: Eco-Friendly Feed Formulation and On-Farm Feed Production as Ways to Reduce the Environmental Impacts of Pig Production Without Consequences on Animal Performance
Source: Front Vet Sci. 2021 Jul 6;8:689012. doi: 10.3389/fvets.2021.689012 (PMC8289902; doi:10.3389/fvets.2021.689012)
Supplement: Supplementary file 1 [file Data_Sheet_1.docx]

**Supplementary Material**

**Table S1** Economic allocation of coproducts applied to the ECOALIM dataset :

| **Ingredients** | **Economic allocation^1^** | **Average price** | **Source** |
| --- | --- | --- | --- |
| Wheat straw | 0% |  |  |
| Wheat flour | 95.90% | 824€/t | USDA 2014 |
| Wheat middlings | 0.76% | 146€/t | La Dépêche Le Petit Meunier |
| Wheat bran | 2.08% | 116€/t | La Dépêche Le Petit Meunier |
| Wheat feed flour | 1.25% | 168€/t | La Dépêche Le Petit Meunier |
| Ethanol from wheat FR | 76.64% | 723€/t | OCDE, prix à la production dans l’UE à 27 |
| DDGS from wheat FR | 23.36% | 222€/t | La Dépêche Le Petit Meunier |
| Ethanol from corn FR | 78.68% | 728.8€/t | OCDE, prix à la production dans l’UE à 27 |
| DDGS from corn FR | 21.32% | 243€/t | La Dépêche Le Petit Meunier |
| Wheat starch | 54.40% | 250€/t | Van Zeist et al, 2012 |
| Wheat gluten | 29.00% | 780€/t | Van Zeist et al, 2012 |
| Wheat gluten feed | 5.00% | 160€/t | Van Zeist et al, 2012 |
| Wheat bran from starch | 8.20% | 120€/t | Van Zeist et al, 2012 |
| Corn starch | 75.75% | 309.8€/t | USDA 2014 |
| Corn gluten feed | 8.78% | 96€/t | USDA 2014 |
| Corn gluten meal | 8.32% | 390€/t | USDA 2014 |
| Corn oil | 7.15% | 851€/t | USDA 2014 |
| Soybean oil | 38.50% | 781€/t | Oil World Annual 2012 |
| Soybean meal | 61.50% | 295€/t | Oil World Annual 2012 |
| Sunflower oil | 83.40% | 1093 US$/t | Oil World Annual 2012 |
| Sunflower meal | 16.60% | 237 US$/t | Oil World Annual 2012 |
| Rapeseed oil | 78.30% | 1191.7US$/t | Oil World Annual 2012 |
| Rapeseed meal | 21.70% | 251.7US$/t | Oil World Annual 2012 |
| Sugar beet | 73.10% | 410€/t | USDA 2014 |
| Molasses beet | 6.60% | 279€/t | USDA 2014 |
| Sugar beet pulp | 20.30% | 132€/t | USDA 2014 |

DDGS = Dried distillers grains with solubles

^1^ Economic allocation is calculated as the ratio of the yield times the price of the co-product, to the sum of the yields times the prices for all co-products.

**Table S2** Nutritional constraints (%) used to formulate pig diets

| **Phase** | **Grower** | | **Finisher** | |  |
| --- | --- | --- | --- | --- | --- |
| **Constraint** | **Min.** | **Max.** | **Min.** | **Max.** |  |
| **Nutritional characteristics** |  |  |  |  |  |
| Net energy (MJ/kg) | 9.8 | 9.85 | 9.8 | 9.85 |  |
| Crude fibre |  | 5.5 |  | 6 |  |
| Crude protein | 15 | 15 | 13.5 | 13.5 |  |
| SID^1^ lysine | 0.82 | 1.23 | 0.72 | 1.08 |  |
| SID methionine | 0.25 | 0.37 | 0.22 | 0.32 |  |
| SID methionine+cysteine | 0.49 |  | 0.43 |  |  |
| SID threonine | 0.5 | 0.76 | 0.45 | 0.67 |  |
| SID tryptophan | 0.15 | 0.22 | 0.13 | 0.19 |  |
| SIS valine | 0.53 | 0.8 | 0.47 | 0.7 |  |
| Calcium | 0.65 | 0.75 | 0.6 | 0.7 |  |
| Total phosphorus |  | 0.48 |  | 0.44 |  |
| Digestible phosphorus^2^ | 2.3 |  | 2.1 |  |  |
| Sodium | 0.15 | 0.25 | 0.15 | 0.25 |  |

^1^SID : Standardized ileal digestible

^2^Apparant total tract digestible

**Table S3** Constraints on incorporation rates of feed ingredients (%) in the Control-diet

| Ingredients | Growing  Minimum rate | Growing  Maximum rate | Finishing  Minimum rate | Finishing  Maximum rate |
| --- | --- | --- | --- | --- |
| Barley | 0 | 7 | 0 | 7 |
| Corn | 0 | 20 | 0 | 20 |
| Oat | 0 | 0 | 0 | 0 |
| Sorghum | 0 | 0 | 0 | 0 |
| Triticale | 0 | 10 | 0 | 10 |
| Wheat | 0 | 35 | 0 | 35 |
| Peas | 0 | 10 | 0 | 10 |
| Faba bean | 0 | 0 | 0 | 0 |
| Corn gluten feed | 0 | 0 | 0 | 0 |
| DDGS from corn | 0 | 0 | 0 | 0 |
| DDGS from wheat | 0 | 0 | 0 | 0 |
| Wheat bran | 0 | 0 | 0 | 0 |
| Wheat gluten feed | 0 | 0 | 0 | 0 |
| Wheat middlings | 0 | 5 | 0 | 5 |
| Sugar beet pulp | 0 | 0 | 0 | 10 |
| Rapeseed oil | 0 | 0 | 0 | 0 |
| Rapeseed meal | 0 | 1 | 0 | 1 |
| Sunflower meal | 0 | 2 | 0 | 2 |
| Soybean meal | 0 | 100 | 0 | 100 |
| L-lysine HCl | 0 | 100 | 0 | 100 |
| DL-methionine | 0 | 100 | 0 | 100 |
| L-threonine | 0 | 100 | 0 | 100 |
| L-tryptophane | 0 | 100 | 0 | 100 |
| Valine | 0 | 100 | 0 | 100 |
| Monocalcium phosphate | 0 | 100 | 0 | 100 |
| Sodium chloride | 0 | 100 | 0 | 100 |
| Calcium carbonate | 0 | 100 | 0 | 100 |
| Trace elements and mineral premix | 0.05 | 0.05 | 0.05 | 0.05 |
| Phytase | 0 | 0.02 | 0 | 0.02 |

DDGS = Dried distillers grains with solubles

**Table S4** Constraints on incorporation rates of feed ingredients (%) in the Eco-diet

| Ingredients | Growing  Minimum rate | Growing  Maximum rate | Finishing  Minimum rate | Finishing  Maximum rate |
| --- | --- | --- | --- | --- |
| Barley | 0 | 30 | 0 | 30 |
| Corn | 0 | 30 | 0 | 30 |
| Oat | 0 | 15 | 0 | 15 |
| Sorghum | 0 | 20 | 0 | 20 |
| Triticale | 0 | 30 | 0 | 30 |
| Wheat | 0 | 30 | 0 | 30 |
| Peas | 0 | 20 | 0 | 30 |
| Faba bean | 0 | 10 | 0 | 10 |
| Corn gluten feed | 0 | 20 | 0 | 20 |
| DDGS from corn | 0 | 20 | 0 | 20 |
| DDGS from wheat | 0 | 20 | 0 | 20 |
| Wheat bran | 0 | 20 | 0 | 20 |
| Wheat gluten feed | 0 | 20 | 0 | 20 |
| Wheat middlings | 0 | 20 | 0 | 20 |
| Sugar beet pulp | 0 | 100 | 0 | 100 |
| Rapeseed oil | 0 | 100 | 0 | 100 |
| Rapeseed meal | 0 | 15 | 0 | 15 |
| Sunflower meal | 0 | 100 | 0 | 100 |
| Soybean meal | 0 | 100 | 0 | 100 |
| L-lysine HCl | 0 | 100 | 0 | 100 |
| DL-methionine | 0 | 100 | 0 | 100 |
| L-threonine | 0 | 100 | 0 | 100 |
| L-tryptophane | 0 | 100 | 0 | 100 |
| Valine | 0 | 100 | 0 | 100 |
| Monocalcium phosphate | 0 | 100 | 0 | 100 |
| Sodium chloride | 0 | 100 | 0 | 100 |
| Calcium carbonate | 0 | 100 | 0 | 100 |
| Trace elements and mineral premix | 0.05 | 0.05 | 0.05 | 0.05 |
| Phytase | 0 | 0.1 | 0 | 0.1 |

DDGS = Dried distillers grains with solubles

**Table S5** Constraints on incorporation rates of feed ingredients (%) in the Local-diet

| Ingredients | Growing  Minimum rate | Growing  Maximum rate | Finishing  Minimum rate | Finishing  Maximum rate |
| --- | --- | --- | --- | --- |
| Barley | 0 | 30 | 0 | 30 |
| Corn | 0 | 30 | 0 | 30 |
| Oat | 0 | 0 | 0 | 0 |
| Sorghum | 0 | 0 | 0 | 0 |
| Triticale | 0 | 10 | 0 | 10 |
| Wheat | 0 | 30 | 0 | 30 |
| Peas | 0 | 20 | 0 | 30 |
| Faba bean | 0 | 10 | 0 | 10 |
| Corn gluten feed | 0 | 0 | 0 | 0 |
| DDGS from corn | 0 | 0 | 0 | 0 |
| DDGS from wheat | 0 | 0 | 0 | 0 |
| Wheat bran | 0 | 0 | 0 | 0 |
| Wheat gluten feed | 0 | 0 | 0 | 0 |
| Wheat middlings | 0 | 0 | 0 | 0 |
| Sugar beet pulp | 0 | 0 | 0 | 0 |
| Rapeseed oil | 0 | 0 | 0 | 0 |
| Rapeseed meal | 0 | 5 | 0 | 0 |
| Sunflower meal | 0 | 0 | 0 | 0 |
| Soybean meal | 0 | 0 | 0 | 0 |
| L-lysine HCl | 0 | 100 | 0 | 100 |
| DL-methionine | 0 | 100 | 0 | 100 |
| L-threonine | 0 | 100 | 0 | 100 |
| L-tryptophane | 0 | 100 | 0 | 100 |
| Valine | 0 | 100 | 0 | 100 |
| Monocalcium phosphate | 0 | 100 | 0 | 100 |
| Sodium chloride | 0 | 100 | 0 | 100 |
| Calcium carbonate | 0 | 100 | 0 | 100 |
| Trace elements and mineral premix | 0.05 | 0.05 | 0.05 | 0.05 |
| Phytase | 0 | 0.02 | 0 | 0.02 |

DDGS = Dried distillers grains with solubles

**Table S6** Environmental global impacts of the average diets, according to the relative contribution of growing and finishing diets to total feed intake (per kg of feed)

|  | **Control-diet** | **Eco-diet** | **Local-diet** |
| --- | --- | --- | --- |
| CC (g CO_2_-eq) | 489 | 367 | 339 |
| NRE (MJ) | 5.08 | 4.56 | 3.07 |
| AC (molc H+-eq) | 0.0094 | 0.0078 | 0.0074 |
| EU (g PO_4_^3-^-eq) | 4.0 | 3.6 | 4.0 |
| LO (m²year) | 1.41 | 1.40 | 1.66 |
| PD (g P) | 3.5 | 2.3 | 2.9 |

CC = climate change; NRE = non-renewable and fossil energy demand; AC = acidification; EU = eutrophication; LO = land occupation; PD = P demand
